# Editorial: Nutrition and Metabolism in School-Age Children

**DOI:** 10.3389/fnut.2022.899126

**Published:** 2022-04-28

**Authors:** Francois-Pierre Martin, Jonathan Pinkney, Jose M. Saavedra

**Affiliations:** ^1^H&H Group, Geneva, Switzerland; ^2^Peninsula Medical School, University of Plymouth, Plymouth, United Kingdom; ^3^Johns Hopkins University School of Medicine, Baltimore, MD, United States

**Keywords:** child, adolescent, nutrition, brain, behavior, development, malnutrition, obesity

Middle childhood and adolescence, the time between 5 and 19 years of age, is a transformative period in the lifecycle. These “school-age years” bridge early life and adulthood through significant and specific physiologic, somatic, cognitive, and psychosocial bursts of change. The pubertal transition in this period marks a major point of inflection and sex-related divergence, expressed by rapid changes in body size, composition, neurocognitive functions, and body systems, including the neuroendocrine axis, cardiovascular changes, skeletal growth, and bone mineralization. It is thus a nutrition-sensitive window, where a complex interplay between genetics and environmental factors, particularly nutrition, play a central role in defining this transformation and its long-term impact on the rest of an individual's life (1, 2).

Today, this age group represents 1.8 billion individuals or one-quarter of the world's population. However, knowledge and research on the nutritional situation of these children is grossly lacking and is the lowest of any age group (3). This Issue of Frontiers in Research on Nutrition and Metabolism in School-Age Children intends to bring attention to some aspects of nutrition during middle childhood and adolescence, including the current situation, long-term effects and consequences, and methodological research challenges, as we gain knowledge in this area.

Over the last 30 years, remarkable global progress has been made in decreasing undernutrition for children of all ages. While global data for this age group is scarce, recent studies shed light on the current situation. The absolute number of underweight children 5–19 years of age peaked around 2000 and has since been decreasing. The prevalence of moderate and severe underweight is minimal in high-income countries, while it remains close to 30% in some Asian countries. Similar gains have been made toward decreasing stunting, and today, the global median height of 5–12 year olds is at or above the WHO median (4, 5). However, rates of stunting, particularly in adolescent girls, remain as high as 52% in Guatemala and 44% in Bangladesh (6).

On the other end of the nutrition spectrum, over the last 40 years, obesity has increased in every country in the world. From 1975 to 2016, the global prevalence of obesity in 5–19 year olds increased eight-fold, from 5 to 50 million girls and from 6 to 74 million boys. More recently, from 2000 to 2016, the proportion of overweight 5–19 year old children rose from 1 in 10 to almost 1 in 5 (7), and Southern African countries had the greatest rise in obesity (about 400% per decade), albeit starting from a very low prevalence (4). Lastly, even though the data for micronutrient deficiencies, so-called “hidden hunger” is even more scant, it is clear deficits of iron, vitamin A, vitamin D, zinc, iodine, and folate remain very high globally (8). For all ages and in all settings, all forms of malnutrition can coexist. It is common to find under- and overnutrition in the same country, the same community, the same household, even in the same child.

The persistence of undernutrition and the explosion of overnutrition and its consequences in later life have led to a concurrent burden of under- and overnutrition, the vast majority of which is borne by low- and middle-income countries (LMIC) and by low-income populations within high-income countries, further accentuating global gaps and disparities in nutrition.

In this issue, Khan et al. add to the understanding of this landscape with an analysis of prevalence of nutritional status and dietary intake of 5–15 years school-going children and adolescents in Pakistan. Using data from 51 studies, they estimate the prevalence of 25% underweight, 23% stunting, 24% wasting, 11% overweight, and 6.9% obese children. These findings are associated with a relatively high intake of carbohydrates, sugar-sweetened beverages, and a low intake of protein-rich foods, fruits, and vegetables. It confirms the “nutritional transition” that LMICs are undergoing, while still exhibiting local cultural pressures, and gender differences. As in most surveys today, overweight was associated with indicators of lower social and economic status.

Studies on diet and nutrition also highlight the need for better and more standardized methodological approaches, including better predictors of weight and adiposity and better assessment of nutrient intake and diet composition. The paper by Wang et al. assesses triponderal mass index compared to BMI as a potentially effective predictor of overweight, metabolic syndrome, and cardiovascular risk in school-age children. Assessing dietary intake and patterns remains critical in identifying gaps and targets for improvement. However, dietary measurement has its own methodological challenges (9). The paper by Jones et al. assesses the reporting of dietary intake using data from a birth to 13 years of age British cohort. It highlights difficulties related to the psychosocial and behavioral changes that characterize this life period, such as the influence of sex and body image. They find high levels of underreporting of energy intake, positively associated with dissatisfaction with their weight, and perception of overweight or obesity, and negatively associated with underweight. Underreporting also had a differential effect on their estimated food and macronutrient intakes. Thus, dietary intake assessments in this age group require correction for these biases and better measurement methods to overcome methodological gaps.

School-age is also a period of brain growth and increase in brain efficiency, critical to the development of executive functions and social and behavioral changes. In this issue, Dmitrichenko et al. show an association between food-approach eating behaviors, especially enjoyment of food and food responsiveness, and brain morphology in adolescence, suggesting that the longer-term eating behavior-brain links are manifest by adolescence. Attaining nutrition literacy during this period may thus have a major impact, as exemplified by the paper of Zeng et al., showing nutrition literacy in middle schoolers varies significantly by socioeconomic status, and plays an important role in determining healthy dietary habits and nutrition.

Lastly, nutrition in school-age years is a period of major acceleration in bone growth and mineral accrual. Total body bone mineral content accrual plateaus by 18–20 years, after which bone mineral content begins to decline (10). School-age is thus a final period “investment” in bone health to decrease the risk of osteoporosis and fractures in later life. In this issue, Abrams reviews the short and long-term effects of nutritional intake on bone health outcomes and the current controversies related to dietary and public health recommendations to support bone health during school age.

The ongoing pandemic of Coronavirus disease is likely to have short- and long-term effects on child nutrition globally, which have yet to be assessed. Regardless, differences and disparities will remain. The effort to improve the nutrition of school-age children will require attention to multiple factors, including economic and social changes, education, cultural barriers, and changes in our food systems, which are intimately linked to the threat of climate change. As stated in UNICEF's Nutrition Strategy for 2020–2030, “nutrition during middle childhood and adolescence is both a right and a window of opportunity for growth, development, and learning, particularly for girls, and for breaking the intergenerational cycle of malnutrition.”

## Author Contributions

All authors contributed to the draft of the manuscript, read, and approved the submitted version.

## Conflict of Interest

F-PM is an employee of H&H Group and was previously an employee of Société des Produits Nestlé SA. The remaining authors declare that the research was conducted in the absence of any commercial or financial relationships that could be construed as a potential conflict of interest.

## Publisher's Note

All claims expressed in this article are solely those of the authors and do not necessarily represent those of their affiliated organizations, or those of the publisher, the editors and the reviewers. Any product that may be evaluated in this article, or claim that may be made by its manufacturer, is not guaranteed or endorsed by the publisher.
